# 原发性浆细胞白血病生物学的研究进展

**DOI:** 10.3760/cma.j.issn.0253-2727.2022.07.014

**Published:** 2022-07

**Authors:** 群 李, 春艳 孙, 豫 胡

**Affiliations:** 华中科技大学同济医学院附属协和医院血液科，武汉 430022 Department of Hematology, Union Hospital, Tongji Medical College, Huazhong University of Science and Technology, Wuhan 430022, China

浆细胞白血病（plasma cell leukemia, PCL）是一种罕见的恶性浆细胞疾病，据国外报道，PCL每年的粗发病率约为0.04 /10万，在浆细胞肿瘤中的占比不足1％[1]，其侵袭性极强，接受传统化疗患者的中位总生存（OS）期不足1年[2]。目前认为，满足外周血中循环浆细胞（circulating plasma cells, CPCs）比例≥20％和（或）CPCs计数≥2×10^9^/L即可诊断为PCL[3]。近年来，有些学者认为该标准过于严苛，CPCs≥5％甚至≥2％的多发性骨髓瘤（MM）患者与传统定义的PCL患者具有相似的预后[4]–[6]。根据有无MM病史可将PCL分为原发性PCL（pPCL）和继发性PCL（sPCL）[2]。虽然都具有高水平的CPCs和极差的预后，但pPCL被认为是一种“从头开始”的临床病理类型，与代表MM终末阶段的sPCL的发生机制迥然不同。本文将从免疫表型、基因组、表观遗传调控、转录组方面对pPCL的生物学特征和发病机制进行探讨，为这一罕见恶性血液肿瘤的预后评估和靶向治疗提供新思路。

一、临床特征

pPCL的中位发病年龄约为61岁，较MM患者约低10岁[7]，其进展迅猛，从疾病起源到表现出临床症状的时间极短，确诊后一个月内死亡率极高，可能与急性白血病的特性有关[8]。pPCL的中位骨髓浆细胞百分比明显高于MM，由于被大量间变性和浆母细胞形态的肿瘤细胞浸润，骨髓造血能力极度匮乏，患者表现出更严重的贫血和出血相关症状[9]–[11]。与MM相比，pPCL较少出现骨质破坏，但更易形成髓外病灶[12]，在体格检查中常发现肝、脾、淋巴结肿大，软组织浆细胞瘤，与肺部浸润相关的胸腔积液及与中枢神经系统浸润相关的神经功能障碍等[2]。实验室指标如血清乳酸脱氢酶和β_2_-微球蛋白水平升高提示pPCL存在更高的肿瘤负荷[9]–[11],[13]。此外，轻链型患者在pPCL中更常见，部分解释了肾功能不全的高发生率，pPCL中非分泌亚型患者的比例也高于MM[7]。

二、免疫表型特征

恶性浆细胞表面的抗原分子不仅可作为肿瘤标志，还参与了肿瘤增殖和抗凋亡、迁移和归巢、免疫逃逸和耐药等生物学行为。

Guikema等[14]发现，CD27抗原在pPCL细胞表面呈现高表达状态，与配体CD70结合后可抑制自发性及地塞米松诱导的细胞凋亡。随后证明该效应是由P38和ERK1/2 MAPK通路介导的[15]。Katodritou等[16]也在3例具有细胞遗传学不良因素的pPCL患者中发现CD27抗原高表达，这些患者均对硼替佐米联合地塞米松治疗敏感，可能是由于硼替佐米对ERK1/2的抑制作用部分抵消了CD27触发的地塞米松耐药效应。Paiva等[17]指出，CPCs表面整合素分子（如CD11a、CD11c、CD29、CD49d、CD49e）及黏附分子（如CD33、CD56、CD117、CD138）的表达明显下调，他们认为CPCs可能起源于骨髓克隆性浆细胞的一个特殊亚群，由于对骨髓微环境的黏附性降低，因此获得了更强的外周血侵犯能力。除了参与黏附，整合素分子LFA-1（CD11a/CD18）还是机体抗肿瘤免疫中的重要刺激因子[18]，pPCL细胞表面LFA-1分子的缺如有助于肿瘤的免疫逃逸[19]。趋化因子及其受体介导了MM细胞的归巢并有助于其在骨髓微环境中留存，然而趋化因子受体CCR1、CCR2和CXCR4在pPCL细胞表面往往不表达，增加了恶性浆细胞在外周血液循环中积累的可能性[20]。

三、基因组特征

1. 染色体异常：pPCL患者出现染色体异常的相对频率较MM患者高，表明pPCL具有更不稳定的基因组。

既往研究指出，半数以上MM患者存在超二倍体核型，相比之下，pPCL中超二倍体少见，大部分患者为复杂的亚二倍体或假二倍体核型[12]。

涉及免疫球蛋白重链基因座（IGH）14q32的染色体易位在pPCL中更为普遍[12],[21]，可能与非超二倍体核型有关[22]。其中t（11;14）（IGH/CCND1融合基因）的发生率最高[12],[23]–[25]，与pPCL中CD20、CD23表达增加和CD56表达降低密切相关[26]–[27]。t（11;14）在MM中属于标准风险指征，但由于携带该易位的pPCL患者常伴随TP53基因突变或双等位基因失活[25],[28]，故此类患者的预后特征还需进一步评估。maf易位t（14;16）（IGH/c-MAF融合基因）和t（14;20）（IGH/MAFB融合基因）在pPCL中出现得更频繁，作为Maf转录因子家族的成员，c-MAF/MAFB通过驱动一系列下游基因的表达发挥致癌作用[29]，但尚不清楚此过程是否介导了MM和pPCL中不同的致癌事件。Chang等[30]报道t（4;14）是影响PCL患者生存的独立危险因素，易位可导致MMSET和FGFR3基因表达上调，已知在MM中，约30％的t（4;14）患者仅存在MMSET基因过表达，遗憾的是，由于大多数针对pPCL的研究未注明FISH探针的类型，目前尚无法判断哪种基因主导了pPCL中t（4;14）的致癌事件。17p的缺失被认为是MM的晚期事件，仅在10％的MM患者中出现，而pPCL中检测到17p缺失患者高达35％，TP53基因随着17p的缺失而丢失，影响G_1_/S期检查点的功能，是促进pPCL基因组不稳定的重要因素。此外，在pPCL中，13q缺失是一种普遍现象，1q扩增/获得和1p缺失的发生频率也显著高于MM患者[25]。表1详细总结了近年来有关pPCL细胞遗传学的研究。

**表1:** 原发性浆细胞白血病细胞遗传学相关研究

作者	年份	国家	样本量（例）	细胞遗传学检查方法	是否分选CD138^+^浆细胞	常见染色体异常发生率（％）						
						t(11;14)	t(14;16)	t(4;14)	del(17p)	del(13q)	1q扩增/获得	1p缺失
Pagano等[11]	2011	意大利	41	FISH	未知	20	0	0	7	46	−	−
Avet-Loiseau等[31]	2012	法国	70	FISH	未知	25	17	21	20	65	−	−
Mosca等[23]	2013	意大利	23	FISH	是	39	30	13	35	74	48	38
Royer等[24]	2016	法国	32	CNAs分析、FISH	是	50	16	6	28	59	53	16
Jung等[32]	2017	韩国	42	FISH	未知	14	10	19	21	29	−	−
Jurczyszyn等[33]	2018	欧美	65	FISH	未知	20	20	20	34	48	17	−
Yu等[13]	2020	中国	37	FISH	是	24	3	0	27	38	30	−
Schinke等[28]	2020	美国	23	FISH	是	22	35	9	−	−	74	−
Cazaubiel等[25]	2020	法国	96	未知	未知	51	14	11	30	−	53	24

注：CNAs：拷贝数异常；−：无数据

2. 基因突变：与MM相比，pPCL的基因突变负荷更高，突变模式更复杂。Cifola等[34]首次对pPCL患者进行了全外显子测序，识别出分布在1 643个编码基因上的1 928个非沉默突变，突变基因主要富集在钙黏素/Wnt信号、细胞外基质-受体相互作用、细胞外基质组成以及G_2_/M细胞周期检查点功能通路中。Lee等[35]通过靶向测序发现pPCL与MM的突变谱重合较少，少数重合的基因如TP53和DIS3在pPCL中的突变更为频繁[34]；其他大部分突变为pPCL所特有，主要影响了DNA损伤修复和细胞信号传导通路[35]。Schinke等[28]发现pPCL每个样本的非同义突变数量（中位数为99）显著高于MM（中位数为64），该队列携带maf易位［t（14;16）或t（14;20）］的患者高达43％，APOBEC突变标记被认为是导致MM中maf易位组患者出现更高突变负荷的原因[36]，但该突变标记在上述队列的pPCL患者中并不显著[28]，故Schinke等认为APOBEC驱动的突变在pPCL发病中的参与度较低，进一步强调了MM和pPCL间生物学机制的差异。对新诊断的pPCL患者进行突变谱分析有助于揭示基因功能改变对肿瘤发生的影响，但由于pPCL具有较高的异质性，其突变谱及突变特征还需通过更多大样本队列进一步总结。

四、表观遗传学特征

许多复杂且相互依赖的表观遗传修饰也参与了pPCL的发生与发展。

Todoerti等[37]揭示了pPCL基因组整体低甲基化的特征，并指出不同的DNA甲基化模式与染色体畸变类型和DIS3基因突变有关，主要影响了参与骨代谢、细胞迁移、转录调控或DNA损伤反应的基因。与MM相比，pPCL低甲基化的基因主要介导了细胞黏附和迁移等过程；部分差异甲基化的基因同时被MM与pPCL的差异表达谱所识别[38]，说明DNA甲基化是造成基因差异表达的重要因素。

此外，组蛋白修饰也是pPCL致癌机制中的重要表观遗传学事件。锌指蛋白19（PHD finger protein 19, PHF19）是多梳抑制复合体2（polycomb repressive complex 2, PRC2）的重要辅助因子，可募集PRC2并激活PRC2的酶促亚基EZH1/EZH2，促进PRC2对靶基因H3组蛋白27位赖氨酸的三甲基化修饰（H3K27me3），导致转录抑制[39]–[40]。Ren等[41]证明在MM和PCL中，PHF19对于维持H3K27me3结构域、保证PRC2发挥基因沉默作用是必需的，这些靶基因包括细胞周期抑制因子和与抗肿瘤免疫密切相关的干扰素-JAK-STAT信号通路分子。PHF19高表达的MM患者即使在序贯双次移植的治疗方案下仍表现出较差的预后[41]，与MM相比，新诊断的pPCL患者PHF19表达水平更高[28]，或许预示了更糟糕的结局。因此，PHF19有望成为pPCL有力的风险标志和潜在的治疗靶点。

五、转录谱特征

更不利的基因组背景似乎并不足以解释pPCL的侵袭程度，转录谱分析为pPCL区别于MM的独特生物学特征提供了更加充分的证据。

1. 基因表达谱：Usmani等[42]发现了203个在pPCL和MM中差异表达的基因，其中一些基因如CD14、TRAF2和CCL2在正常情况下主要在单核细胞和巨噬细胞中表达，揭示了骨髓瘤细胞的髓样分化与白血病表型之间的关联。Cazaubiel等[25]指出，与MM相比，pPCL过表达的基因主要富集于MYC靶标和G_2_/M检查点通路，而低表达的基因主要在P53、缺氧和TNF-α信号通路中发挥作用，这些通路可成为研究两种疾病不同致癌机制的切入点。Todoerti等[38]对接受来那度胺和地塞米松作为一线治疗的pPCL患者进行转录谱分析，发现YIPF6、EDEM3、CYB5D2三个基因可鉴别出对治疗反应不佳的患者，另有27个基因与预后较差显著相关；此外，503个基因的表达与MM存在差异，其中有26个基因的表达趋势反映了浆细胞肿瘤的恶性进展。

最近，Rojas等[43]对具有相似细胞遗传学特征（均存在17p缺失，其他细胞遗传学的差异无统计学意义）的pPCL和MM患者进行了转录谱分析，旨在探索统一遗传背景条件下两种疾病在基因表达方面的差异。结果表明，尽管拥有相似的细胞遗传学背景，转录水平的差别仍可鉴别pPCL和MM [43]。其中，剪接体相关组分的表达在pPCL和MM中存在显著差异，由于剪接体可影响细胞内mRNA的剪接模式，因此他们进一步在1 249个总体表达无差异的基因中发现了2 727个差异表达的异构体，突出了选择性剪接事件在pPCL发病机制中的作用，强调了异构体水平的转录谱分析对于pPCL生物学研究的特殊意义。

2. 非编码RNA表达谱：以lncRNA和miRNA为代表的调节性非编码RNA可在不同层次调控蛋白质编码基因的表达，影响肿瘤细胞内的各种生物学过程。

Lionetti等[44]鉴定出pPCL中存在一系列与MM差异表达的miRNA，其中表达上调的主要是一些具有“致癌”效应，或在免疫应答、造血中发挥作用的miRNA，如miR-155、miR-21和miR-17-92家族等，有趣的是，位于13q31.3的miR-17-92家族在pPCL中的显著过表达并不受到13q缺失状态的影响；而一些被证明有抑癌作用的miRNA在pPCL中的表达更低，如miR-663、miR-193b、miR-126及let-7a等[44]。根据已报道的功能和靶基因预测，这些差异表达的miRNA可能在pPCL的发生发展中起到推动作用。

此外，Ronchetti等[45]在PCL患者（包含24个pPCL和12个sPCL）中发现了85个与正常人相比表达异常的lncRNA。他们还进一步探索了PCL中lncRNAs和miRNAs之间的交叉调控，发现lnc-MCL1-2可以作为miR-17家族的海绵，调控维持肿瘤生长的MCL1基因的转录；lnc-AGBL1-4和miR-185-5p之间也可能存在某种环路，最终导致癌基因NTRK3的表达失控[46]。对非编码RNA之间相互作用的挖掘将有助于深入了解pPCL的基因调控网络。

六、小结与展望

pPCL是一种恶性程度极高、预后极差的浆细胞肿瘤，与MM相比，其发病年龄轻、病情进展迅猛、早期死亡率高。近年来，关于pPCL生物学背景的研究不断从免疫学、基因组、表观遗传学、转录谱等方面为pPCL细胞脱离骨髓微环境、获得比MM更强的侵袭性提供证据，然而囿于样本数量的限制，pPCL的疾病特征依旧难以准确归纳。未来需借助多中心合作的力量开展更大规模的前瞻性队列研究，采用更加精准的高通量组学技术如异构体表达谱测序、蛋白质谱分析等深入探寻pPCL与MM克隆性浆细胞的生物学差异，及时构建实验模型验证相关致病机制。除了继续开展两种疾病的横向对比，还应对pPCL自身的纵向发展进行追踪，以确定更多与预后相关的标志物，识别出敏感和耐药群体的分子特征，开发出更加有效的靶向治疗策略。
